# Co-Designing a Web-Based, Gamified, Auditory–Cognitive  Dual-Task Training System for Older Adults with Hearing Loss

**DOI:** 10.3390/healthcare13222926

**Published:** 2025-11-15

**Authors:** Ivy Yan Zhao, Tsz Wai Lau, Chen Li, Janet Ho-Yee Ng, Eleanor Holroyd, Robert Sweetow, Engle Angela Chan, Angela Y. M. Leung

**Affiliations:** 1 School of Nursing, The Hong Kong Polytechnic University, Hong Kong SAR, China; yan-ivy.zhao@polyu.edu.hk (I.Y.Z.);; 2 WHO Collaborating Centre for Community Health Services, School of Nursing, The Hong Kong Polytechnic University, Hong Kong SAR, China; 3 Research Institute for Smart Ageing (RISA), The Hong Kong Polytechnic University, Hong Kong SAR, China; 4 Department of Applied Social Sciences, The Hong Kong Polytechnic University, Hong Kong SAR, China; 5 Department of Computing, The Hong Kong Polytechnic University, Hong Kong SAR, China; 6 Speech Therapy Unit, Department of Language Science and Technology, The Hong Kong Polytechnic University, Hong Kong SAR, China; 7 International and Engagement Office of the Dean, Auckland University of Technology, Auckland 1010, New Zealand; 8 Department of Otolaryngology, Head and Neck Surgery, School of Medicine, University of California, San Francisco, CA 94115, USA

**Keywords:** age-related hearing loss, cognitive function, gamified training system, assistive technologies, co-design

## Abstract

**Background:** Age-related hearing loss (ARHL) is associated with decreased communication, reduced social engagement, cognitive decline and an increased risk of dementia globally. Although increasing studies report the benefits of combing auditory and cognitive training for older adults with ARHL, more evidence is needed to examine its effects. Moreover, existing training programs have been developed with minimal end-user involvement leading to low adherence rates. This study aimed to investigate the role of co-design in the development of an auditory–cognitive training system for older adults with ARHL. **Methods:** A co-design methodology was employed. Digital recordings of the co-design workshops were transcribed verbatim. An established reflexive thematic analysis methodology was used. **Results**: Fifteen older adults with ARHL, referred to as “co-researchers”, participated in three co-design workshops until data saturation was achieved. Consultations were held with two key service providers. Three key themes emerged: (1) older adults with ARHL prefer a user-friendly auditory–cognitive training system; (2) clear, localized and colloquial instructions for the training tasks are necessary; and (3) diversified, tailor-made and dual-task training tasks, performed in an interactive and game-like mode, can motivate and sustain usage of the training system. As a result, a prototype of a web-based, gamified, and adaptive auditory–cognitive dual-task training system was co-designed. **Conclusions**: Our findings affirmed the importance of genuinely listening to the voices of end-users and creating a system that is responsive to their needs and preferences. Future study is recommended to examine the effects of this system on older adults with ARHL.

## 1. Introduction

Age-related hearing loss (ARHL) is a gradual, progressive and bilateral symmetric sensorineural hearing loss (HL) that is associated with aging [1]. ARHL is a highly prevalent and under-recognized disability [2]. According to the World Report on Hearing, there were around 70% of people over the age of 70 (>8.3 m people) with HL internationally [3]. In China, around 60% of individuals over 60 experience ARHL [4]. The percentage is higher in Hong Kong, with 82.5% reported in 2021 [5]. In a recently published study, over two-thirds (67.5%) of older adults with ARHL had not been formally diagnosed in Hong Kong [6]. A lack of knowledge about ARHL was identified as a major cause of low diagnosis and treatment [7].

Numerous empirical studies, systematic reviews and meta-analyses have shown that ARHL is associated with cognitive decline and greater risk of diagnosing with dementia [8,9,10]. Lower scores on tests of memory and executive function were more likely to be reported by older adults with greater HL [11,12]. The results of cohort studies have demonstrated that a mild level of ARHL could also increase the long-term risk of cognitive decline and dementia [13,14,15,16]. While social engagement and depressive symptoms have been identified as mediators in the relationship between ARHL and cognitive status [17], Kiely et al. [18] also suggested that cognitive impairment could be independently associated with lower levels and accelerated declines in peripheral hearing ability.

Evidence indicates that both cognitive and auditory abilities influence speech perception in older adults. Auditory training involves active engagement with sounds to improve auditory processing, while cognitive training employs mental exercises to maintain or enhance core cognitive abilities [19]. The Neuroplasticity Theory posits that auditory–cognitive training harnesses neuroplasticity through targeted exercises that stimulate the brain, potentially enhancing auditory processing and cognitive functions [20]. Effective auditory training for older adults with HL requires integrated sensory–cognitive approaches, with the synergistic integration of auditory processing and working memory serving as the core mechanism driving perceptual–cognitive improvements [21].

Training interventions for older adults with HL increasingly emphasize personalization, adaptability, and interactivity to improve everyday listening abilities [22]. For example, a UK randomized controlled trial (RCT) employed computer-delivered phoneme training with eleven synthesized phoneme continua from real voice recordings, employing a three-interval, three-alternative, forced-choice oddball paradigm. The training significantly reduced hearing disability and improved attention and working memory in older adults with mild HL, with benefits lasting at least four weeks post-training [23]. Conversely, the online Listening and Communication Enhancement (LACE) training developed in the United States, which focus on improving various aspects of auditory processing skills like speech comprehension, auditory memory, and the ability to distinguish speech from background noise, showed limited cognitive benefits but significantly improved hearing ability in older adults with lower levels of HL [24]. However, no auditory or auditory–cognitive training is available in Chinese, introducing a cultural bias. Further research is needed to identify which training tasks are most effective and determine whether gains generalize to broader cognitive functions, which remains uncertain.

Given the central nervous system’s plasticity, even in old age, brain plasticity-based techniques can help restore diminished hearing and cognitive functions [25]. Dual-task training, which involves performing two tasks simultaneously or sequentially, has demonstrated significant cognitive benefits for older adults with HL [26]. A Japan-based RCT [27] indicated that auditory–cognitive dual-task training resulted in changes in the regional gray matter volume of participants. More research is needed to examine the effects of auditory–cognitive training and improve dual-task abilities in individuals with ARHL [28].

Moreover, existing training programs have often been developed with minimal end-user involvement, resulting in low adherence rates. For instance, a study suggested that adherence to an auditory–cognitive training schedule can be a challenge for participants at home without supervision [25]. Involving end-users in the development process helps identify potential usability issues early on, allowing for adjustments that enhance the overall user experience [29]. Therefore, this study aimed to investigate the role of co-design in the development of an auditory–cognitive training system with older adults with ARHL, who are the potential end-users. The co-design process, key results, and discussions are presented in the subsequent sections.

## 2. Materials and Methods

### 2.1. Research Design

**Figure 1:**
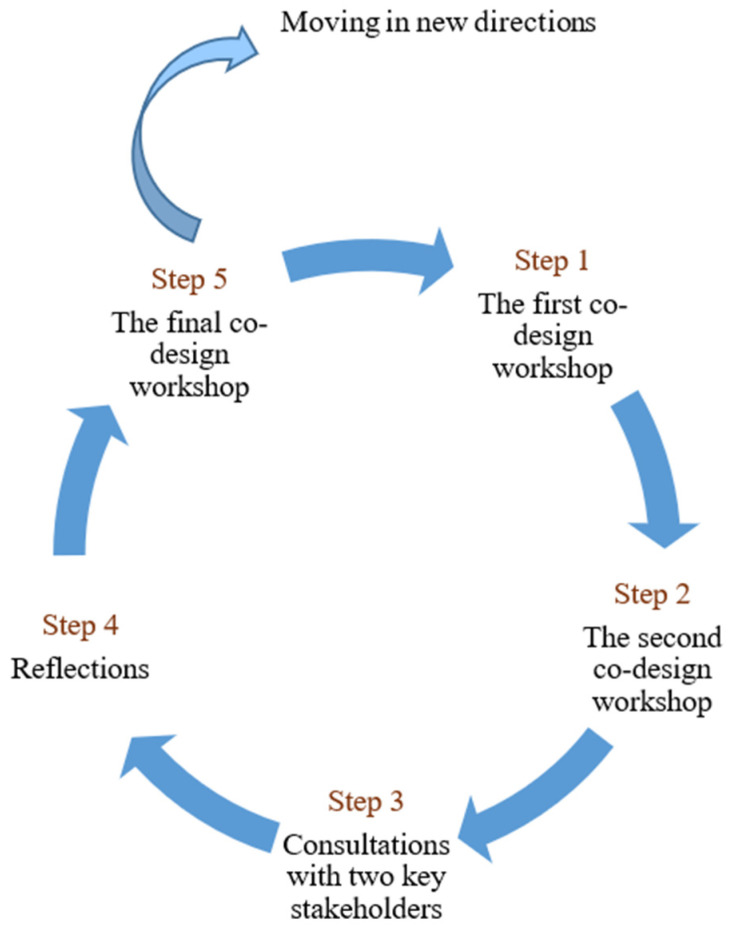
The community-based participatory research cycle was used to guide the co-design workshops at the Hong Kong Polytechnic University from 5 January to 2 May 2024.

A co-design methodology was employed to develop and conduct co-design workshops and consultations with community service providers. A five-step community-based participatory research cycle (Figure 1) [30] guided the data collection process for this study. We developed this cycle based on McNiff and Whitehead’s action–reflection cycle [29] and tested in a previous co-design study [30]. The action–reflection cycle, which includes the steps of planning, acting, observing, and reflecting, allows participants to continuously refine their practice based on evidence and reflection [29]. We adapted this model to shift the focus from action to a more participatory approach within community settings [30], which enabled participants (called co-researchers) and researchers to collaborate on an interactive enquiry to address the research aim and answer research questions [30,31]. Co-researchers, being part of the community, are more likely to reflect the actual needs and priorities of older adults with ARHL. This research method not only enhances the cultural acceptability for the community members but also empowers co-researchers by valuing their experiential knowledge and involving them in the research process [32]. Consistent with these principles, this approach facilitated a collaborative and interactive data collection process, valuing the knowledge, sharing, and creative ideas contributed by co-researchers [33]. This paper adheres to the Consolidated Criteria for Reporting Qualitative Research (COREQ) checklist [34].

The research team prepared the workshop by leveraging co-researchers’ expertise in the field and considering cultural and age-specific expectations, values, and behaviors. Two co-design workshops were conducted first to continuously develop, review, and refine the concept of an auditory–cognitive training system and a paper-based prototype for older adults with ARHL. In the subsequent step, two key stakeholders, Christian Family Service Centre and Hong Kong Sheng Kung Hui, were engaged to provide feedback on how the findings from the co-design workshops could be further implemented in the community services. Based on complete feedback from previous steps, a web-based prototype of an interactive and adaptive auditory–cognitive training system was developed, tested, and reviewed by co-researchers, and their feedback was collected to refine the system further. Below, we will detail each step and elaborate on the rationale behind the design and implementation choices of the study.

### 2.2. Co-Researchers and Recruitment for a Co-Design Study

Advertisements (in Chinese) were distributed to local community groups, non-governmental organizations (NGOs), and shared networks during June to December 2023. Additionally, snowball sampling was employed to recruit hard-to-reach subjects, such as older adults living in isolation. Individuals interested in participating in the study were directed to contact the study team. Purposive sampling was adopted for study recruitment. Demographic information was collected following informed consent procedures. A selection framework (Table 1) was created to include a diverse group of co-researchers with different genders, ages, educational and occupational backgrounds. The inclusion criteria for co-researchers were as follows: (1) ethnic Chinese aged 60 years and above, (2) mild to moderate hearing loss, diagnosed by a pure-tone average of 20 to 50 dB across octave frequencies from 0.5 to 4 kHz in both ears; (3) self-reported normal hearing in younger age; and (4) provision of informed consent to participate in the study. The exclusion criteria were as follows: (1) a diagnosis of a major neurocognitive disorder; and (2) serious medical or psychiatric illness (e.g., severe depression, schizophrenia, bipolar disorder) or a visual impairment that would interfere with participation.

### 2.3. Ethical Considerations

This study was approved by the Human Subjects Ethics Sub-Committee of the Hong Kong Polytechnic University (Reference number: HSEARS20230223006). Informed consent was obtained from all co-researchers prior to their involvement in the study, ensuring they were fully aware of the research objectives and their rights. To protect privacy and confidentiality, all study data have been de-identified, ensuring that individual co-researchers cannot be traced back to their data. Co-researchers were compensated for their time and contribution to the research with supermarket coupons valued at USD 200; HKD ≈ 26 USD. No identifiable images of individual co-researchers are included in the manuscript or Supplementary Materials.

### 2.4. Preparing the Co-Design Workshops

All co-design workshops were conducted face-to-face at the Hong Kong Polytechnic University. The venue for the workshop was selected to ensure accessibility and comfort. The meeting room was quiet and private, with available restrooms and flexible setup and layout options. The room was equipped with working desks, flip chart space, electronic facilities for visual presentations and a table for food and drinks, all aimed at providing a welcoming and inclusive environment. Two tables were positioned together in the center of the room, each equipped with a digital recording device. Name cards displaying the initials of the co-researchers were placed on the table to designate seating arrangements, ensuring that all co-researchers had a clear view of the AV screen. Additionally, two side tables were set up on one side of the room to facilitate small group discussions. Throughout the workshops, co-researchers had the ability to review and update information on the charts as needed.

Each of the three workshops was structured to last approximately 2.5 to 3 h to minimize co-researcher fatigue and ensure engagement. Author 2 facilitated the workshops under the supervision of Author 1. During the co-design workshops, some guiding questions and instructions were provided to help co-researchers engage with both paper-based and online prototypes and offer feedback. A list of these facilitating questions is included as Supplementary Document S1. All the workshops were audio recorded. Notes written on flip charts and post-it notes by the co-researchers were collected. We observed and documented the process by which the co-design workshops were implemented and reflected on the findings and flow of the workshop after its completion. Issues arising were discussed regularly within the research team, and relevant strategies were developed for the subsequent steps.

### 2.5. Step One: The First Co-Design Workshop

The first co-design workshop was held on 5 January 2024. The workshop was divided into two sections. The first section was opened by the facilitators (Authors 1 and 2) with a welcome speech and a round of introductions. The facilitators then generated ground rules with co-researchers, helping to establish a democratic process. The author 2 explained the steps of the co-design process, presented an overview of existing interventions aimed at improving listening abilities and cognitive functions for older adults with ARHL, and introduced the concept of co-designing an auditory–cognitive training system. The purpose of this introduction was to provide co-researchers with background information about the research and facilitate their engagement in the ensuing discussion. The associated responsibilities of the co-researchers were also outlined.

**Figure 2:**
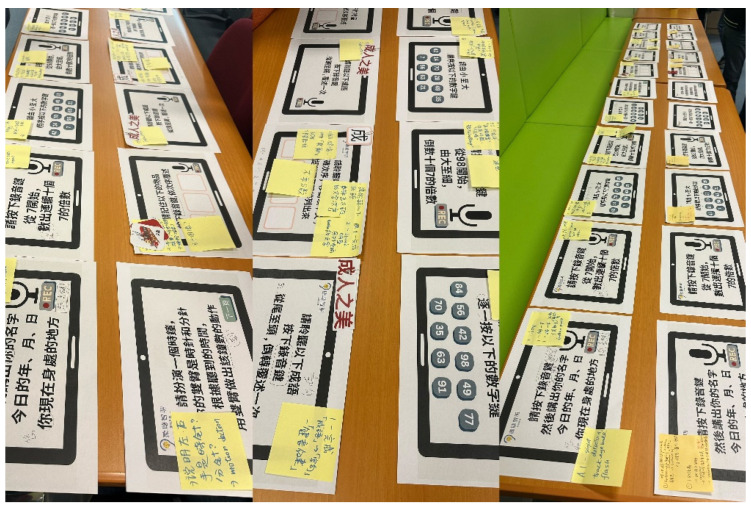
Paper prototypes of the auditory training and cognitive training content in Chinese, such as identifying the current year, today’s date and current location, performing calculations, and listening to sentences, were developed by the study team. Feedback from co-researchers during the co-design workshop was collected and captured on post-it notes.

In the Section 2, paper-based prototypes of the auditory–cognitive training system were provided to the co-researchers. These prototypes, designed to mimic the user interface (Figure 2) by the Jockey Club Design Institute for Social Innovation at the Hong Kong Polytechnic University, included separate components of auditory training and cognitive training, which were derived from our previous systematic review. Speech-in-noise training, rapid speech training, and competing speaker training were identified as effective methods for improving listening skills in noisy environments [35], speech perception [36], speech comprehension, and the ability to focus on and understand the target voice [37] for individuals with hearing loss. Audio recordings from a hearing training module were played for them, featuring short, medium, and long sentences set against a noisy background, at rapid speed, and with multiple speakers. Participants wore headsets and adjusted the volume to a comfortable level, and no hearing difficulties were reported. Moreover, training tasks aimed at enhancing executive functions, perceptual–motor or visuospatial skills, memory, and attention have been shown to effectively increase the global cognitive function of older adults [38]. Co-researchers were divided into two groups, each facilitated by Author 1 and Author 2, to review the prototypes and audio recordings incorporating the aforementioned training components. They conducted group discussions and recorded their comments on the paper prototypes. At the end of the workshop, all the paper prototypes with comments from the two groups were laid out for a final review by the co-researchers.

### 2.6. Step Two: The Second Co-Design Workshop

The second co-design workshop was undertaken on 19 February 2024. Co-researchers were invited to review the revised paper prototypes and audio recordings, which had been updated based on the feedback from the first co-design workshop. They were tasked with designing the details of each training task, including the wording of task instructions, colors, sounds, animations, task duration, and methods as well as the design of dual-task training in order to gamify each training task. Co-researchers used sticky notes to add their ideas and suggestions to each paper prototype.

### 2.7. Step Three: Consultations with Key Stakeholders

In order to translate the co-designed auditory–cognitive system into practice, consultations were held with two stakeholders: Christian Family Service Centre and Hong Kong Sheng Kung Hui. These stakeholders were chosen because they have integrated the WHO Integrated Care for Older People (ICOPE) framework [39] into their routine healthcare services, making hearing loss assessment a regularly part of their operations for older adults. Individual meetings were arranged with these stakeholders. During these meetings, the background and feature functions were presented, and they were provided with a testing account to experience the training modules.

### 2.8. Step Four: Reflections

**Figure 3:**
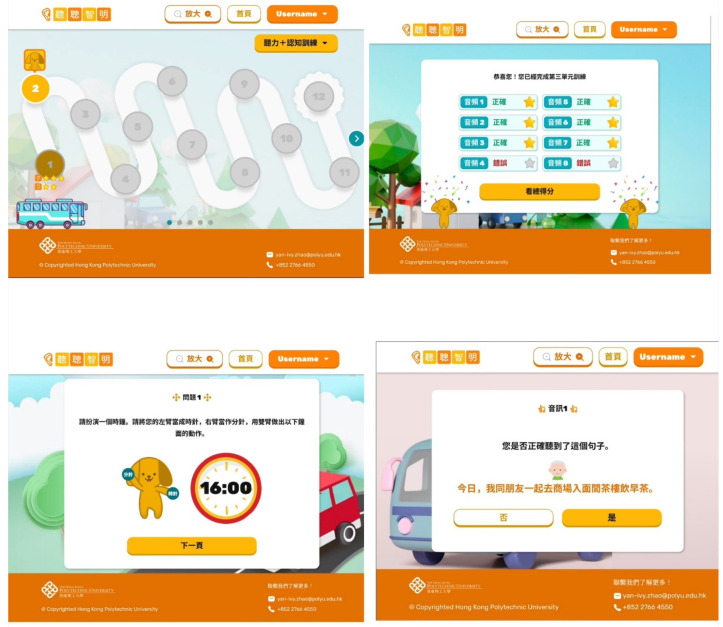
Screenshot examples of a web-based auditory–cognitive training system prototype developed by the study team. They illustrate the daily training pathway, the scoring system, a cognition task that asks users to indicate a time using hand gestures, and a listening task presented with background noise.

Two stakeholders’ feedback and suggestions on the training system idea co-designed by co-researchers in the previous workshops were collected. A participant referral process was collaboratively discussed. A summary was subsequently sent to co-researchers through WhatsApp or email for further input. After integrating all the feedback from steps one to four, the research team transferred the paper prototypes and audio recordings to an online format and created a web-based prototype of the auditory–cognitive training system (see Figure 3).

### 2.9. Step Five: The Final Co-Design Workshop

The final co-design workshop was held on 2 May 2024. Co-researchers were invited to engage with the web-based prototype and practice using the training system in a real-world context. This system incorporates sound effects and interactive elements, such as likes, praises, fireworks, preferred color, and leaderboards to enhance the engagement. This approach offers older adults with ARHL the flexibility of individual training while also fostering a sense of group participation through the leaderboard. Approximately 60 min were used for completing an auditory–cognitive training module prior to the facilitated discussion. Co-researchers then discussed their experiences with navigating the system, the layout, the speed of loading exercises, and provided further suggestions during a group discussion.

### 2.10. Data Analysis

Digital recordings of the co-design workshops were concurrently transcribed verbatim. The transcripts were then returned to the co-researchers for verification to ensure the accuracy of the content. Any necessary changes were made to confirm that the findings were shaped by the participants rather than researcher bias or interest. Data were analyzed using an established reflexive thematic analysis methodology, a six-step process to effectively organize and interpret qualitative data integrated [40]. This method incorporates reflection on the co-researchers’ perspectives and roles in the research process and demonstrates the stability and consistency of the research process over time. We used both inductive and deductive reasoning, applied latent coding. For example, co-researchers’ preferences and perceptions on auditory–cognitive training system were explored, and codes were generated directly from the data without preconceived categories. Meanwhile, some codes related to co-designing specific features were created based on literature about hearing and cognitive training. All members of the research team reviewed codes and came to a consensus on what the final themes should be.

## 3. Results

### 3.1. Reflections on the Workshop Experience

Thoughtful preparation of the co-design workshops can foster trust among the co-researchers and establish a strong relationship with them. Many co-researchers were unaware of their hearing issues before joining this study and lacked knowledge about the risk of HL contributing to cognitive decline. Their general awareness about hearing health was low, so more background information was provided by the study team to help them understand the issue and prepare them for co-designing the system. Due to the lack of available hearing services, the co-researchers were proactive in participating in the co-design workshops, eager to contribute to improving existing services. They also affirmed that this process is crucial for co-designing an auditory–cognitive training program for older adults with ARHL.

### 3.2. Findings from the Co-Design WORKSHOPS

Initially, sixteen candidates consented to participate, but one withdrew due to having other commitments at the time of the scheduled workshops. A total of fifteen co-researchers participated in the study (Table 2). They were 4 males and 11 females, aged from 60 to 76, who had been identified as having mild to moderate HL with a pure-tone average of 20 to 50 dB across octave frequencies 0.5 to 4 kHz in both ears.

Three key themes were generated: (1) older adults with ARHL prefer a user-friendly auditory–cognitive training system; (2) clear, localized and colloquial instructions for the training tasks are necessary; and (3) diversified, tailor-made, dual-task training tasks, performed in an interactive and game-like mode, can motivate and sustain usage of the training system. As a result, a prototype of a web-based, interactive and adaptive auditory–cognitive training system was co-designed in this study (Figure 3).

#### 3.2.1. Theme 1: Older Adults with ARHL Prefer a User-Friendly Auditory–Cognitive Training System

Most co-researchers agreed that a digital training system should be easy for older adults to operate, and that even basic features should be clearly explained. For instance, co-researcher 14 (61 years, F) suggested that the research team create a short introduction video: “*The video can serve as a strategy guide. In the video, the basic features can be introduced.*” This sentiment was echoed by co-researcher 13 (62 years, F), who stated, “*The research team should provide us with a technical support hotline*”.

Many older adults experience decreased eyesight or visual problems. Font size and color could improve readability, attract their attention, and enhance their engagement. Co-researcher 4 (71 years, M) suggested, “*The system font should use bright colours and bold text to create high contrast with the white background, thereby attracting participants’ attention*.” Additionally, older adults may not be familiar or remember the flow of training tasks, so instruction wording and icons should be clear. As co-researcher 8 (69 years, F) recommended, “*I suggest adding an arrow icon button beside the ‘Record’ button to subtly indicate to users that they can click the button to begin recording*.”

Furthermore, older adults often take on different roles and responsibilities at home, such as looking after grandchildren or serving as caregivers for an older spouse. A training system with a flexible access approach is preferred. A web-based training system was highly preferred by most co-researchers. They expressed a desire for the training to be accessible via their mobile phones. As co-researcher 15 (75 years, F) mentioned, “*It would allow me to engage with the training at home or even while sitting in a park*”. Co-researcher 1 (60 years, F) echoed this statement, suggesting, “*I recommend offering individual, family, and group editions. The family edition can encourage older couples to use it together, while the group edition can be tailored for senior centres or audiology clinics, allowing the system to be adapted to various environments and needs*”.

#### 3.2.2. Theme 2: Clear, Localized and Colloquial Instructions for the Training Tasks Are Necessary

All the co-researchers critically reviewed each training task instruction and agreed that the Cantonese language should be localized and colloquialized to make it more understandable. For example, as co-researcher 5 (61 years, M) stated, “*I recommended replacing ‘count out 數出’ with ‘speak out 講出’ in the instructions, as it is more idiomatic and easier to understand. Additionally, ‘count out 數出’ could be misinterpreted to mean that something needs to be counted or listed.*” Co-researcher 6 (60 years, F) also commented “*I recommended revising the task instruction for mimicking a clock to ‘Listen to the time and use both arms to demonstrate the corresponding clock face movements’. Replace ‘hour 鐘數’ with ‘clock face 鐘面’ in the instruction to make it more understandable by local older adults*”.

Secondly, they considered that instructions should be clear and straightforward as some older adults may have difficulty understanding and following multiple tasks. Several co-researchers suggested that “*In the task of role-playing as a clock, the instructions should clearly state the left hand represents the hour hand and the right hand represents the minute hand, as this can be a common source of confusion for older adults*” (Co-researcher 5, 61 years, M, and co-researcher 6, 60 years, F, and co-researcher 12, 63 years, F). Moreover, “*The task instruction ‘Listen to the idioms and then sort them out’ should be changed to ‘Please listen to the following sentences, move the following words, and arrange them in the correct order’. The purpose of this statement is to clearly instruct the task requirement*” (co-researcher 1, 60 years, F, and co-researcher 2, 72 years, M).

#### 3.2.3. Theme 3: Diversified, Tailor-Made, Dual-Task Training Tasks, Performed in an Interactive and Game-Like Mode, Can Motivate and Sustain Usage of the Training System

For the training tasks, it was agreed that no more than an hour per training module would be appropriate for older adults to maintain engagement and prevent fatigue. All co-researchers concurred on a structure consisting of a 5 min preparation period, 25 min of auditory training, 25 min of cognitive training, and a 5 min wrap-up. All co-researchers found using Chinese idioms and poetry interesting. However, some suggested diverse training topics and contents, noting that “*Contents for training should be fresh every day*” (co-researcher 5, 61 years, M, and co-researcher 7, 66 years, F). Co-researcher 13 (62 years, F) added, “*If the contents are repeated, people might recall the content from their previous training session, rather than engaging real training*”.

For both the hearing and cognitive training exercises, co-researchers expected the difficulty levels to be adaptive to meet their diverse training needs. They suggested that users should be given the opportunity to adjust the volume for a comfortable listening level at the beginning of the training. This volume setting should be saved to ensure the quality of the training experience. Incorporating challenges of dual-task training could be more appealing for users with better hearing and cognitive function. All the co-researchers preferred a sequential dual-task design, where auditory training is followed by cognitive training. This approach was considered to meet the training needs without being too demanding for older adults. Co-researcher 5 (61 years, M) suggested “*The counting numerical tasks can be designed to gradually increase in difficulty, starting with multiples of 2, 4, and 5, then moving on to multiples of 6 and 9, and finally to multiples of 7, to ensure that participants do not get frustrated by tasks that are too challenging*”. Co-researcher 6 (60 years, F) and 12 (63 years, F) recommended, “*We can provide different difficulty levels (easy version & advanced version), incorporating dual-task trainings. Because the participants of the training system are older people, and their educational background or hearing abilities may vary, having different difficulties allows participants to choose the one that suits their own level. Dual-task trainings can be more challenging and attractive for those who have maintained good hearing or cognitive capabilities*”.

All the co-researchers agreed that the training system should be designed to be more interactive, enjoyable, and interesting, empowering older adults to continue engaging. For example, co-researchers 4 (71 years, M) and 6 (60 years, F) suggested, “*I recommend implementing a scoring system to motivate participants, such as displaying a score of 100, and if they meet the criteria, they will level up to the next stage, encouraging them to continue training. It would be beneficial to provide communication tips and strategies, allowing users to gain useful knowledge after completing the training module*”. Co-researcher 15 (75 years, F) added, “*A reward system can be used to incentivize participants to keep training. For instance, if they complete three days, they will earn one stamp, and if they finish the remaining two days, they will get an extra stamp, bringing their total to two. Once they’ve accumulated enough, they can redeem them for rewards of small gifts*. *It is like young people playing electronic games; setting up a leaderboard can encourage participants to persist in completing their training*” co-researcher 6 (60 years, F) suggested, “*If the answer is correct, the system should display a congratulatory message, motivating older people to continue*”. Co-researcher 4 (71 years, M) recommended, “*Given the research team’s emphasis on daily training, I suggest that the system sends notification as reminders to participants’ devices or tablets, prompting them to complete their daily training and providing a motivational boost*”. Co-researcher 12 (63 years, F) added, “*I suggest that the system provides audio feedback for correct answers to encourage participants to keep answering correctly. Additionally, consider using emoticon symbols, like a smiley face for correct answers and a sad face for incorrect ones. This will make the experience more enjoyable and interactive, and participants will be more likely to stay engaged and complete the remaining tasks”.*

### 3.3. Consultation Outcomes

First, the prevalence of ARHL is high in Hong Kong, but the available service in the community is very limited. Both stakeholders agreed on the importance of addressing HL in older adults at an earlier stage to prevent further cognitive decline. As a result, they have made hearing loss assessment a regular part of their operations for older adults, but they currently lack a solution. They confirmed their plans to integrate this training system into their general healthcare pathways, ensuring that older adults with HL will be referred to this training system. Second, both stakeholders found the system design to be age-friendly and appreciated the incorporation of cultural components. Based on their suggestions, the interface was made adjustable to various digital devices, ensuring users can access the auditory–cognitive training via their mobile phones, as not every older adult has a tablet or carries one while traveling. This training system can be easily managed by the older adults themselves, which can reduce the labor burden for the service providers.

## 4. Discussion

### 4.1. Principal Findings

This study demonstrated the feasibility of using a co-design research approach with older adults with ARHL. Their engagement as co-researchers enabled them to collectively identify hearing healthcare needs and co-design a web-based auditory–cognitive training system tailored for older adults with ARHL. Our findings are consistent with those of other studies [41], which have shown that a co-design approach helps identify potential barriers and challenges that may have been overlooked. Additionally, it provides practical recommendations to enhance the user-friendliness, feasibility, and acceptability for end-users [41].

### 4.2. Comparison to Prior Work

Involving participants as co-researchers in co-designing the system extends previous studies that focused primarily on the issue of HL and cognitive decline [22]. This study highlights the importance of clear, localized, and colloquial instructions for the training tasks to meet the specific needs of older adults. The training tasks in our program involve a significant amount of repetition and “drill” work. To motivate and sustain engagement with training tasks among older adults with HL, co-researchers recommended diversified, tailor-made, and interactive training tasks. Our findings are consistent with previous studies suggesting that the training process should be engaging and entertaining, as many exiting programs tend to be tedious [42]. An engaging training program may lead to better compliance rates. Conversely, with less engaging programs, participants may not pay full attention while completing tasks, especially if the tasks require significant effort [43]. Our findings revealed contradictions in the participants’ views and needs. On one hand, they wanted simple, easy-to-use interfaces, but on the other hand, they desired more signs and icons pointing to relevant buttons. This confirmed the necessity of our intervention to incorporate technology that meets the specific needs of users. Previous technology-based interventions have also suggested that incorporating more interaction, visualizations, and a game-like environment could motivate older participants and enhance adherence to the intervention [44]. In this study, the opinions of key stakeholders were considered. They reassured the co-researchers that the issues they had raised had been heard by service providers, demonstrating that the co-designed training system will be integrated into their community health service delivery.

Conducting co-design workshops with older co-researchers presented some challenges, which had been previously identified by Zhao et al. [30]. These included transportation difficulties, group dynamics, communication skills, conflict with domestic work obligations, and health issues. In the current study, the issue of HL made communication more complicated and challenging, even among co-researches, who had mild to moderate HL and functioned unaided in daily life. It aligns with Baldwin et al.’s findings that hearing problems, visual problems, and breathlessness negatively impacted older co-researchers’ commitment and ability to participate in a co-design research process [45]. To address these challenges, detailed preparation of the co-design workshops was essential. This included providing microphones to help those with HL, using clear and simple language, and offering written materials to supplement verbal communication. Additionally, observing co-researchers’ reactions, establishing ground rules, and double-checking responses were crucial to ensuring the quality of this study. Nonetheless, the advantages of involving participants as co-researchers far outweighed these difficulties. The involvement of the co-researchers resulted in a plan that is more closely aligned with the real needs of people than one that the researchers could have developed on their own. The findings from this co-design study will inform future research and intervention development direction.

### 4.3. Future Directions

Early management of ARHL and cognitive decline in older adults is more cost-effective than delayed treatment [46]. Preliminary evidence has demonstrated the beneficial effects of managing ARHL on cognitive function through auditory and cognitive training [47]; however, more well-designed trials are needed to examine its effectiveness, determine which training tasks are most effective, and sustain improvements beyond trained tasks, which are essential to determine the effectiveness of a training intervention [48]. Dual-task interventions incorporating various cognitive exercises are promising and could maximize the potential benefits of lifestyle activities on cognition [48], but the underlying mechanisms need to be further understood. Moreover, poor dual-task performance is suggested as an early indicator of dementia and can greatly impact the activities of daily life [49]. Individuals who perform poorly on dual-task tests (e.g., listening while engaged in cognitively demanding tasks) are also likely to have interpretation and communication problems [49]. Finding ways to improve dual-task ability may reduce functional decline and mediate effective communication for people with ARHL [28]. Because there is no documented auditory–cognitive dual-task training developed for older Chinese adults with HL, and auditory training in a participant’s native language is essential for comprehension, engagement, and listening skills [50], future studies are needed to examine its effects and to generate new knowledge about the associated mechanisms of training and transfer.

### 4.4. Limitations

This study has several limitations. Firstly, these findings are contextualized to Chinese ethnicity. While purposive sampling can ensure that the sample includes individuals with relevant experiences, it may also limit diversity and representativeness of the sample, as individuals who do not meet the specific criteria may have been excluded. Despite the limitations related to ethnicity, the study’s insights into co-designing an auditory–cognitive dual-task training system may still be relevant to other countries. ARHL is a global health issue, so the principles and strategies developed in this study could be adapted and applied in different cultural and geographical contexts. Secondly, balancing the diverse needs and preferences of all co-researchers and stakeholders can be challenging, potentially leading to compromises that may not fully satisfy any single individual. To mitigate this issue, the design process has included multiple rounds of feedback and iteration. By continuously engaging co-researchers and refining the design based on their input, the team aimed to address as many needs as possible. The workshops were conducted to prioritize features and needs, and prototypes were developed and tested with co-researchers to gather real-world feedback. This allowed the team to identify and address issues early in the process. In the future, implementing ongoing feedback mechanisms even after deployment can help identify and address emerging needs or issues. Thirdly, the study involved translating materials from Chinese to English. Even with careful translation efforts, some subtle meanings or cultural nuances might not be fully captured in the English version. This can lead to a loss of important context or depth in the findings, potentially affecting the interpretation and application of the results in non-Chinese contexts.

## 5. Conclusions

This co-design study directly engaged older adults with ARHL and service providers in the creation of a training system. Our findings emphasize the importance of genuinely listening to the voices of end-users and developing a system that is responsive to their needs and preferences. The resulting prototype—a web-based, adaptive, hearing-cognitive dual-task training system—incorporates user-friendly features, clear and localized instructions, and diversified, gamified training tasks. These co-designed elements are expected to enhance user engagement and adherence, addressing a critical gap in the current hearing healthcare services. Future research is needed to examine the feasibility and acceptability of this system among older adults with ARHL, assess their engagement and adherence, and evaluate the effectiveness of this system. This could lead to widespread implementation and significant public health benefits, ultimately reducing the burden of ARHL and its associated cognitive decline.

## Figures and Tables

**Table 1:** A selection framework was used to include a diverse group of co-researchers with different backgrounds.

Key Elements of the Framework	
Define the population	Older adults with mild to moderate ARHL
Key characteristics	Ethnic Chinese Aged 60 years and above Diverse educational and occupational backgrounds Various living situations Mild to moderate hearing loss Permanent residents of Hong Kong, from different regions Able to commit to attending co-design workshops
Selection criteria	The inclusion criteria/exclusion criteria of this study
Recruitment strategy	Advertisements (in Chinese) were distributed to local community groups, non-governmental organizations (NGOs), and shared networks. Snowball sampling was employed to recruit hard-to-reach subjects, such as older adults living in isolation.
Sample size	12–16 participants is an optimal number for co-design workshops, ensuring each participant can engage effectively.
Data collection	Audio recording Flipchart and post-it notes

**Table 2:** Demographic information of the co-researchers (N = 15).

Demographic Variables	Values n (%)
**Age range (Years)**	
60–64	8 (53)
65–69	2 (13)
70–74	4 (27)
>75	1 (7)
**Gender**	
Male	4 (27)
Female	11 (73)
**Educational level**	
Primary	2 (13)
Secondary	7 (47)
Tertiary	6 (40)
**Employment status**	
Employed	2 (13)
Retired	13 (87)
**Marital status**	
Single	1 (7)
Married	13 (86)
Divorced	1 (7)
**Lived alone**	
Yes	2 (13)
No	13 (87)
**Hearing loss (Right ear)**	
Mild (20–40 dB HL)	10 (67)
Moderate (41–50 dB HL)	5 (33)
**Hearing loss (Left ear)**	
Mild (20–40 dB HL)	14 (93)
Moderate (41–50 dB HL)	1 (7)

## Data Availability

The dataset used and analyzed in this study is available from the corresponding author. The data are not publicly available due to privacy reason.

## Supplementary Materials

The following supporting information can be downloaded at https://www.mdpi.com/article/10.3390/healthcare13222926/s1, Document S1: guiding questions for co-design workshops.

## Abbreviations

The following abbreviations are used in this manuscript:

ARHL	Age-related hearing loss
