# Supplementary material for: Co-Designing a Web-Based, Gamified, Auditory–Cognitive Dual-Task Training System for Older Adults with Hearing Loss
Source: Healthcare (Basel). 2025 Nov 15;13(22):2926. doi: 10.3390/healthcare13222926 (PMC12652463; doi:10.3390/healthcare13222926)
Supplement: Supplementary file 1 [file healthcare-13-02926-s001.zip › healthcare-3891995-supplementary.pdf]

Supplementary document S1: guiding questions for co-design workshops

1. What is your experience navigating through the prototype?
2. Which features or elements do you find confusing or difficult to use, and how might they be improved?
3. How do you approach completing the main tasks within the prototype?
4. How do you interpret the instructions or labels provided?
5. In what ways could the information be presented more clearly and concisely?
6. What jargon or terminology, if any, do you find unclear?
7. What are your thoughts on the color scheme used in the prototype?
8. How do the colors influence your perception of important elements?
9. In what ways do the colors affect your overall experience?
10. How do you find the responsiveness of the interactive elements?
11. Which interactive features do you find particularly useful or engaging, and why?
12. What issues, if any, do you encounter with interactive elements, and how do they affect your experience?
13. How would you describe the enjoyment level of your experience with the prototype?
14. What elements contribute to making the interaction more entertaining or enjoyable for you?
15. Which aspects of the prototype do you find most fun, and why?
16. How does the prototype capture and hold your attention?
17. What features encourage you to explore the prototype further?
18. What elements enhance or detract from your engagement with the prototype?
19. How does the prototype address cultural differences in its design and content?
20. What cultural references, if any, do you find inappropriate or confusing?

21. How does the prototype resonate with your cultural background?

22. What suggestions do you have for enhancing the prototype?
